# The effect of caffeic acid phenethyl ester and Ankaferd Blood Stopper on the diabetic and nondiabetic gingival wound healing: an experimental study

**DOI:** 10.3906/sag-2007-193

**Published:** 2021-04-30

**Authors:** Mehmet GÜL1, Serkan DÜNDAR, Gökhan ARTAŞ, Akın YİĞİN, Abdulsamet TANIK, Mehmet Emrah POLAT, Erhan Cahit ÖZCAN7

**Affiliations:** 1 Department of Periodontology, Faculty of Dentistry, Harran University, Şanlıurfa Turkey; 2 Department of Periodontology, Faculty of Dentistry, Fırat University, Elazığ Turkey; 3 Department of Medical Pathology, Faculty of Medicine, Fırat University, Elazığ Turkey; 4 Department of Genetic, Faculty of Veterinary, Harran University, Şanlıurfa Turkey; 5 Department of Periodontology, Faculty of Dentistry, Adıyaman University, Adıyaman Turkey; 6 Department of Oral and Maxillofacial Surgery, Faculty of Dentistry, Harran University, Şanlıurfa Turkey; 7 Department of Plastic and Reconstructive Surgery, Faculty of Medicine, Fırat University, Elazığ Turkey

**Keywords:** Ankaferd Blood Stopper, caffeic acid phenethyl ester, diabetes, gingival wound healing

## Abstract

**Background/aim:**

Healthy wound healing is very important for patient comfort. Diabetes is one of the factors that negatively affect wound healing. Ankaferd Blood Stopper (ABS) and caffeic acid phenethyl ester (CAPE) are antiinflammatory and antimicrobial agents and may have positive effects on wound healing.

**Materials and methods:**

In this study, 72 male Wistar albino rats were used. Rats; control, CAPE, ABS, diabetes + control, diabetes + ABS and diabetes + CAPE groups were divided into 6 groups. A healthy 36 rats created diabetes using streptozotocin (STZ). A gingival wound was created using a 4-mm punch biopsy in the gingival tissue under the lower anterior incisors of the rats.

**Results:**

The comparison between the nondiabetic groups had a statistically significant positive effect compared to the control group of CAPE and ABS (P ˂ 0.05). In the comparison between ABS and diabetes + ABS groups and in the comparison between CAPE and diabetes + CAPE groups, a decrease in vascularization in diabetes + CAPE groups was observed and it was statistically significant (P ˂ 0.005).

**Conclusion:**

ABS and CAPE have been found to have positive effects on gingival wound healing in the nondiabetic group. We think that this situation is caused by its antiinflammatory and antimicrobial properties.

## 1. Introduction

Wound healing in the gums includes the answers that took place in order to close the defects in the oral mucosa. Responses in tissues are important to prevent the spread of pathogens into the tissue and to prevent chronic infection. Inflammatory responses play an important role after injuries, as they occur during wound healing. The wound healing process is seriously affected by aging. After tissue damage, a large number of biological pathways begin to work to prevent infection and restore tissues. Cells that increase in the wound healing process are usually neutrophils, monocytes, lymphocytes, dendritic cells, endothelial cells, keratinocytes and fibroblasts [1].

Diabetes mellitus (DM) is a metabolic disorder characterized by chronic hyperglycemia. It is one of the most important causes of micro and macro complications in patients [2]. Patients with diabetes are more likely to have infections, so periodontal disease is also more likely [3]. Periodontal diseases are gingivitis due to bacterial infection. If this situation progresses, it may cause loss of the alveolar bone, which is one of the tissues surrounding the tooth. In recent years, it has been suggested that there is a direct relationship between diabetes and periodontal disease [4–6]. There is an impairment in the healing process in diabetic patients. Neutrophil responses, fibroblast movements, and angiogenesis are defective. In this case, it affects the wound healing process negatively [7,8].

Caffeic acid phenethyl esters (CAPE) are bioactive materials derived from the honeybee propolis extracts having antioxidant, antiinflammatory and antineoplastic. It is stated that CAPE is also beneficial for wound healing. CAPE has a positive impact on reducing reactive oxygen species (ROS) formed with plasma, lung and kidney lipid peroxidases. It also accelerates the formation of neo-epidermis in incisional lesions. CAPE induces faster wound contraction and new epithelial formation by reducing oxidative damage [9].

Ankaferd Blood Stopper (ABS), widely used as a hemostatic agent in Turkey. The ABS contains *Glycyrrhiza glabra, Vitis vinifera, Alpinia officinarum, Urtica dioica, Thymus vulgaris* [10]. Studies have reported that ABS has strong antimicrobial properties along with its effectiveness in bleeding control [11]. In the studies it was suggested that ABS can be used in dental treatments because of its hemostatic activity and strong antimicrobial properties [12,13].

The aim of this study is to investigate the effects of topical ABS and CAPE on secondary healing of experimental excisional wound areas formed in the gingival mucosa of diabetic and nondiabetic rats.

## 2. Materials and methods

In this study, 72 male Wistar albino rats (mean age: 7 weeks; weight: 250–300 g) were used. To ensure the protection of experimental animals, the recommendations in the Helsinki Declaration were fully followed. Rats were kept at 55% humidity, room temperature 22 ± 2 °C and 12 h light/dark cycle. Rats were kept separately in a controlled room (21 °C and 12 h light/12 h dark cycle) and in cages where they could freely access water and food. Rats were divided into 6 groups; control, CAPE, ABS, diabetes + control, diabetes + ABS and diabetes + CAPE groups. In these six groups, 4-mm mucosal defects were created in gingiva. The rats in the ABS and diabetes + ABS groups were applied topically with 0.2 mL of ABS solution. Rats in the CAPE and diabetes + CAPE groups were applied 50 mmol/kg of CAPE topically. Pressure was applied with a sterile pad until bleeding control was achieved. All treatments were applied for 14 and 21 days (n = 6).

### 2.1. Diabetes process in rats

Streptozotocin is a narrow-spectrum antibiotic with diabetogenic properties and has toxic effects on beta cells of Langerhans islets in the pancreas. It also has a genotoxic effect. Usually, a single dose of streptozotocin (40–65 mg/kg, intraperitoneal) is used to develop diabetes [14]. Streptozotocin (STZ) (Sigma Chemical Co., St. Louis, MO, USA) intraperitoneal injection was performed with 50 mg/kg of 0.2 mL 10 mM citrate solution to 36 healthy rats. One week later, blood glucose levels were measured in rats (Optima blood glucose meter, Hausverwaltung Optima GmbH, Hannover, Germany). If the blood glucose value was 350 mg/dL, the rats were accepted as diabetic [15].

### 2.2. Surgical procedure

**Figure 1:**
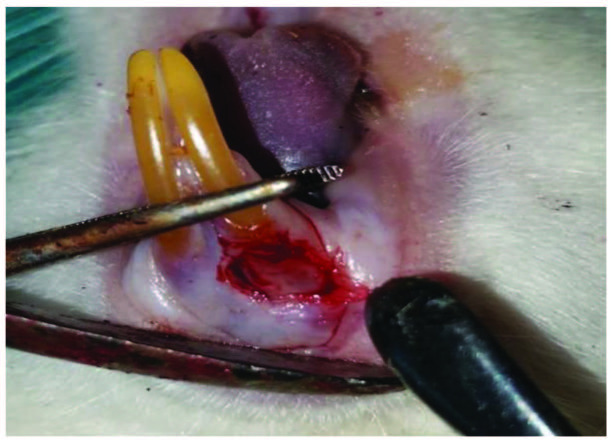
4-mm defect created on gingival mucosa.

The animals were fasted for 8 h before performing surgical procedures and general anesthesia. Surgical procedures were performed under sterile conditions and under general anesthesia. Xylazine hydrochloride (Rompun, Bayer, Leverkusen, Germany, 10 mg/kg) and ketamine hydrochloride (Ketasol, Richter Pharma, Wels, Austria, 40 mg/kg) were used as general anesthetics. General anesthetics were administered intramuscularly with the help of an insulin injector. A full-thickness gingival wound was created using a 4-mm punch biopsy in the gingival tissue under the lower anterior incisors of the rats (Figure 1). Rats are monitored daily for signs of pain, infection, limited movement, anorexia, and weight loss after surgery. Antibiotic (50 mg/kg penicillin) and analgesic (0.1 mg/kg tramadol hydrochloride) were applied i.m. to prevent infection and pain after surgery. They were applied once in every 24 h for 3 days. Subjects were sacrificed on the 14th and 21st days.

After the surgical procedures, the wound area was left to heal. Sacrification was done using sodium thiopentone (lethal dose: 60 mg/kg). The tissue in the wound area was excised together with healthy tissues. Tissues taken were kept in 10% formaldehyde for histological evaluation, while tissues to be analyzed with real-time PCR were kept at –80 °C until analysis time.

### 2.3. Histopathological method

Gingival tissue was directly put in neutral buffered formalin fixative. After complete fixation of the removed tissues, it was kept under running water for 12 h. Then, for the dehydration process, tissues were kept in gradually increasing alcohol series (30%, 50%, 70%, 80%, 90%, 96% and 100%) for 12 h. After the xylol transparency, the tissues were infiltrated and embedded in paraffin blocks. Six-µm sections were obtained from paraffin blocks and stained with hematoxylin-eosin dye for routine dyeing.

**Figure 2:**
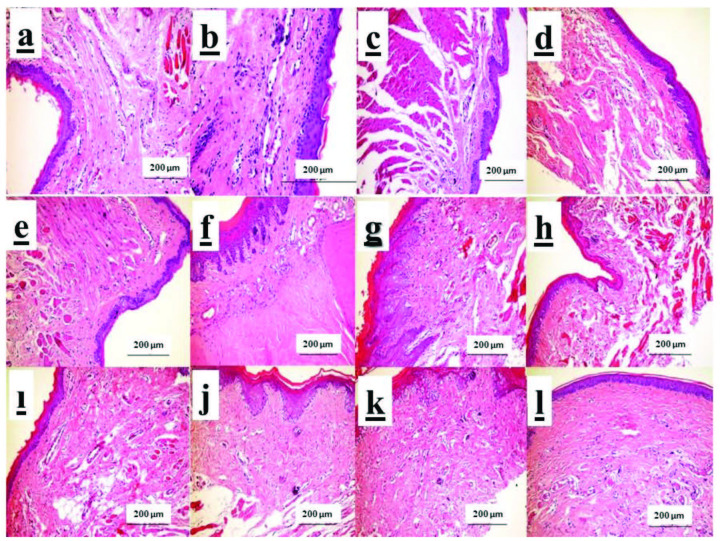
Hematoxylin-eosin staining bar 200 μm: a)14th day ABS group, b)14th day CAPE group, c) 14th day control group, d)21st day ABS group, e)21st day CAPE group, f) 21st day control group, g)14th day diabetes + ABS group, h)14th day diabetes + CAPE group, ı) 14th day diabetes + control group, j) 21st day diabetes + ABS group, k) 21st diabetes + CAPE group, l) 21st diabetes + control group.

Light microscopic evaluation was performed in sections of 6-µm thickness of gingival wound area. Gingival wound healing were evaluated using an image analysis program for inflammation (grading 0–4), epithelialization and vascularization. All images of histological samples were obtained from a digital camera connected to a light microscope. The images were transferred to the computer in original magnification. Histological analysis was performed with the help of Olympus DP71 (Tokyo, Japan) software imaging system (Figures 2a–2l).

### 2.4. RNA isolation, cDNA synthesis and qRT-PCR analysis

Tissue samples were taken into mRNA storage solution and stored at –80 °C. High Pure RNA Tissue Kit Tisssue (Roche 12 033 674 001, Roche Diagnostics, Risch-Rotkreuz, Switzerland) protocol was followed and samples were homogenized and RNA isolation was performed. RNA purity and integrity were checked with a nanodrop device. With the Transcriptor First Standard cDNA Synthesis Kit (Roche 04896866001), cDNA was made according to the procedure [16,17].

**Figure 3:**
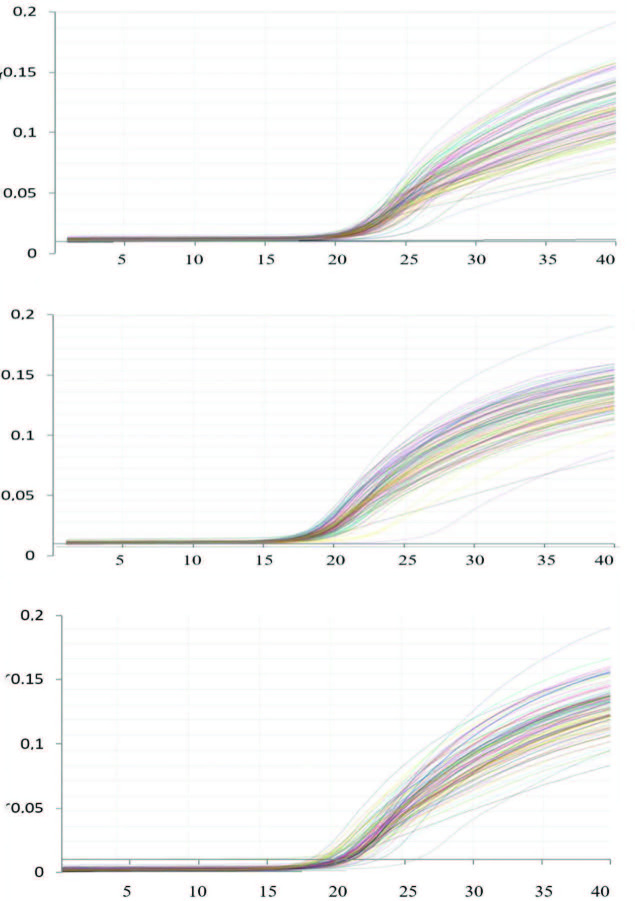
Target genes amplification curves real-time PCR data a) TNF-α, b) IL-6, c) e-NOS.

In the real-time PCR study, the following primer sequences were used for TNF-α, IL-6, e-Nos, and target genes with ACTB (beta actin) reference gene (Table 1). LC DNA Master SYBR Green I kit (Roche 04707516001) was used [17]. PCR mixing protocol real-time PCR (master-mix), 3 µLddH2O, 10 µL LightCycler 480 DNA Master SYBR Green I2x conc, Primer. Forward (Target or ACTB) 1.0 µL, Primary Reverse (Target or ACTB) 1.0 µL, total mix volume 15, µL and 3.0 µL of each Target cDNA on top of this mixture, with a final concentration volume of 18 µL was studied. Real-time PCR protocol, on the other hand, was performed with 30 s cooling at 1 cycle 95 °C, 30 s at 95 °C for 45 cycles, 30 s at 60 °C, 20 s at 72 °C, and 30 s at 1 cycle 40 °C. Working with Lightcycler real-time PCR device. Then, the concentration values determined according to the crossing point (Cp) results of the samples were obtained with the 2-∆∆CT method (Figures 3a–3c).

**Table 1:** Primers base pairs sequence of the expression gens.

Oligonucleotide ID	Nucleotide sequence	Accession
TNF-α	5’- GCAGGTCTACTTTGGAGTCATT -3’	Roy et al., 2018
	5’- GGCTCTGAGGAGTAGACGATAA -3’	
e-NOS	5’- CCGGCGCTACGAAGAATG-3’	Hermawatı et al., 2018
	5’- CAGTGCCACGGATGGAAATT-3’	
IL-6	5’- CCACCAGGAACGAAAGTCAA -3’	Our design
	5’- TCAACAACATCAGTCCCAAGAA -3’	
ACTB	5‘-AGATGACCCAGATCATGTTTGAGA-3’	Yono et al., 2009
	5’-ACCAGAGGCATACAGGGACAA- 3’	

### 2.5. Statistical analysis

Statistical analysis of the data in our study was done using SPSS 20.0 for Windows (IBM Corp., Armonk, NY, USA) statistical program. Histological and PCR data with descriptive statistics; numerical values are shown as mean arithmetic values (M) and standard deviation (SD) values. Wilcoxon test was used for nonparametric data. The normal distribution of the data was evaluated with the Shapiro–Wilk test. Mann–Whitney U test was used to compare the nonnormally distributed data between the two groups and the Kruskal–Wallis test was used for more than two groups. In comparing more than two groups, the Mann–Whitney U test with Bonferroni correction was performed. In all statistical tests, P < 0.05 value was considered significant.

## 3. Results

As a result of histological analysis performed on the 14th day: In the CAPE group, a decrease in inflammation was detected compared to the control group and it was found statistically significant (P = 0.045). In addition, a decrease in inflammation was detected in the ABS group compared to the control group, which was found statistically significant. Also, an increase in inflammation was observed in the diabetes + ABS group compared to the ABS group, which was found statistically significant (P = 0.007). On the 14th day, an increase in epithelialization was observed in the CAPE and ABS group compared to the control group and was found statistically significant (P = 0.014, P = 0.07). No significant difference was observed in epithelialization in the diabetic group compared to the control group. However, in comparison between diabetes + ABS and ABS group and between diabetes + CAPE group and CAPE group, a decrease in epithelialization was found in diabetic groups and it was found statistically significant (P = 0.007, P = 0.006). On the 14th day, vascularization increased in the CAPE group compared to the control group and was found statistically significant (P = 0.041). In addition, in the comparison between ABS and diabetes + ABS groups and in the comparison between CAPE and diabetes + CAPE groups, a decrease in vascularization in diabetes + CAPE groups was observed and it was statistically significant (P = 0.006, P = 0.041, Table 2).

**Table 2:** Histopathological comparison between groups on 14 and 21 days.

	Days	Control	ABS	CAPE	Control+ diabetes	ABS + diabetes	CAPE + diabetes	P* value	Intergroup P value
Inflammation	14	1.67 ± 0.82	0.83± 0.41	0.83 ± 0.41	2.50 ± 0.55	2.17 ± 0.75	2.33 ± 0.82	0.001	0.045a, 0.045b, 0.995c, 0.423d, 0.789e, 0.665f, 0.007g, 0.007h
	21	1.00 ± 0.63	0.67 ± 0.82	0.67 ± 0.52	2.00 ± 0.80	2.00 ± 0.89	2.00 ± 0.89	0.010	0.382a, 0.336b, 0.859c, 0.981d, 0.906e , 0.935f, 0.031g, 0.016h
Intergroup P value		0.150	0.523	0.523	0.299	0.733	0.495		
Epithelisation	14	1.67 ± 0.52	2.67 ± 0.52	3.00 ± 0.63	1.33 ± 0.52	1.33 ± 0.52	1.50 ± 0.55	0.001	0.014a, 0.007b, 0.336c, 0.986d, 0.575e, 0.575f, 0.007g, 0.006h
	21	2.67 ± 0.82	3.33 ± 0.52	3.33 ± 0.82	2.17 ± 0.41	2.17 ± 1.17	1.83 ± 0.41	0.007	0.118a, 0.176b, 0.860c, 0.788d, 0.176e, 0.719f, 0.65g, 0.007h
Intergroup P value		0.031	0.056	0.382	0.018	0.163	0.241		
Vascularization	14	2.67 ± 0.82	3.50 ± 0.55	3.67 ± 0.52	2.17 ± 0.75	2.00 ± 0.63	2.67 ± 0.82	0.004	0.073a, 0.041b, 0.576c, 0.652d, 0.337e, 0.150f, 0.006g, 0.041h
	21	2.17 ± 0.75	2.83 ± 0.75	3.33 ± 0.52	2.33 ± 0.82	1.83 ± 0.41	2.50 ± 0.55	0.014	0.167a, 0.016b, 0.206c, 0.176d, 0.789e, 0.043f, 0.019g, 0.030h
Intergroup P value		0.337	0.116	0.269	0.665	0.598	0.789		

As a result of histological analysis performed on the 21st day: An increase in vascularization was detected in the CAPE group compared to the control group, and it was found statistically significant (P = 0.041). No statistically significant difference was found between other groups (P ˃ 0.05, Table 2).

As a result of the PCR analysis: IL-6 levels were evaluated and no significant difference was found on the 14th day. On the 21st day, a statistically significant difference was found between control and ABS groups, control + diabetes and ABS + diabetes groups and between ABS and ABS + diabetes groups (P ˂ 0.05). TNF-α levels were evaluated and there was no statistically significant difference between the groups on the 14th day (P ˃ 0.05). On the 21st day; a statistically significant difference was found between control and CAPE groups and between ABS and CAPE groups (P ˂ 0.05) (Table 3). e-NOS levels were evaluated and an increase was observed in the groups with ABS and CAPE on the 14th day and was found statistically significant (P ˂ 0.05). In addition, in the analysis performed on the 21st day, an increase was detected in the ABS group compared to the control group and it was found statistically significant (P = 0.016, Table 3).

**Table 3:** Comparison of Tnf- α, IL- 6 and e-NOS with real-time PCR analysis between groups on 14 and 21 days.

	Days	Control	ABS	CAPE	Control+ diabetes	ABS + diabetes	CAPE + diabetes	P* value	P value between groups
PCR IL-6	14	19.69 ± 13.04	16.90 ± 10.48	40.93 ± 8.24	14.85 ± 1.22	17.02 ± 4.79	18.47 ± 5.22	0.869	NS
	21	8.31±4.10	18.80 ± 9.02	16.64 ± 6.68	40.41± 10.14	5.94± 2.54	23.91± 9.41	0.011	0.025a. 0.055b. 0.631c. 0.016d.0.337e. 0.055f. 0.025g. 0.423h
P value between groups		NS	NS	NS	NS	NS	NS		
PCRTNF-α	14	1.74 ± 0.41	1.06 ± 0.21	3.74 ± 1.97	1.77 ± 0.02	3.42 ± 1.45	6.25 ± 2.88	0.312	NS
	21	1.08 ± 0.42	1.33 ± 0.95	2.62 ± 0.84	12.30 ± 5.93	1.15 ± 0.53	5.33 ± 2.61	0.006	0.423a. 0.006b0.030c. 0.078d.0.522e. 0.055f. 0.631g. 0.630h
P value between groups		0.337	0.522	0.748	0.078	0.200	0.749		
PCR e-NOS	14	1.10 ± 0.67	5.33 ± 2.39	8.02 ± 4.84	4.23 ± 1.39	4.96 ± 1.34	8.77 ± 1.22	0.011	0.004a. 0.006b. 0.262c. 0.873d.0.873e. 0.995f. 0.631g. 0.632h
	21	2.91 ± 1.19	7.50 ± 2.51	3.88 ± 2.70	22.22 ± 10.91	2.08 ± 0.66	5.38 ± 2.08	0.031	0.016a. 0.263b. 0.078c. 0.200d.0.873e. 0.200f. 0.006g. 0.522h
P value between groups		0.020	0.262	0.078	0.337	0.037	0.936		

## 4. Discussion

Many factors have negative effects on wound healing at various stages of the wound healing process. These factors disrupt tissue production and delay wound healing [18]. Kido et al., created diabetes by applying Streptozotocin to the rats and formed wounds in the palatal region. They evaluated wound healing that occurred in the wound area. They reported a delay in wound healing on days 7, 14, and 21. They determined the number of subjects used in each group to be 6 [19]. The contraction of the wound begins after the injury and peaks on the 14th day. The degree of contraction of the wound varies depending on the depth of the wound. Up to 40% reduction in the size of the wound occurs in full-thickness wounds. The total amount of collagen increases in the early period of the repair. It reaches its maximum after 14 and 21 days of injury [20].

In our study, rats were made diabetic using streptozotocin. Then a secondary wound was created in 4-mm full-thickness gingiva. The number of subjects was determined as 6 for each group. Vascularization, epithelialization and inflammation were evaluated histologically on the 14th and 21st days. TNF-α, e-NOS and IL-6 was evaluated by PCR analysis method on the 14th and 21st days.

Salles et al. created an 8-mm wound in mice and diabetic mice with streptozotocin. Then, they applied CAPE to the wound area to evaluate the antioxidant and antiinflammatory properties of CAPE in diabetic mice. The diabetes + CAPE group suggested that there was a strong tendency towards more contraction and reepithelialization compared to the diabetes group, and treatment of diabetic lesions with CAPE is a good option for a therapeutic treatment with the benefit of being a natural source product that can reduce the wound healing complications of diabetes [21]. Magro-Filho and de Carvalho reported that CAPE’s topical application of wound surfaces reduces inflammation in oral cavity surgery [22]. Armutcu et al. have reported that CAPE exhibits strong antioxidant, antiinflammatory and wound healing properties due to nuclear factor-κB inhibition [23].

Similarly, in our study, a decrease in inflammation, an increase in epithelialization and vascularization was observed on day 14 between the CAPE-treated nondiabetic group and the nondiabetic control group, and it was found statistically significant. Among the comparison between diabetes-treated groups, there was a statistically significant difference in the CAPE-treated groups on the 14th day, although inflammation decreased, epithelialization and vascularization increased. On the 21st day, there was no significant difference in inflammation and epithelialization, however, a statistically significant increase in vascularization was observed in the CAPE group compared to the control group.

Topal et al. evaluated effects of ABS and silver sulphadiazine (SSD) cream on burn wounds on days 0, 7, 14, 21, 28. All rats in the ABS and SSD groups were completely covered by granulation tissue and epithelized, however, the wounds in the control group were not epithelialized completely. On the 7th day, mean wound areas that did not heal and mean wound contraction rates did not show a significant difference between the groups. They reported that the mean percentage of wound contraction in the ABS and SSD groups was significantly higher at 14, 21 and 28 days than in the control group. They reported that, wound healing is characterized by a decrease in neutrophil count and an increase in vascular count, and that ABS, as well as SSD, can be successfully used in burn wound healing [24]. In a study by Akalin et al., they created a full-thickness wound defect and applied ABS. They examined the contraction percentage histopathologically and reported that the ABS had a positive effect on wound healing [25]. Pamuk et al. suggested that ABS induces an increase in soft tissue healing by stimulating angiogenesis and vascular endothelial cell function in the treatment of periodontal defects using autogenous cortical bone graft. They also reported that it prevented gingival recession and thus increased clinical attachment gain [26]. Keceli et al. reported that the application of medical herbs positively contributed to soft tissue healing [27].

Similarly in our study, ABS was applied on the 14th and 21st days. On day 14, a decrease in inflammation, an increase in epithelialization and vascularization was observed compared to the control group, and it was found statistically significant. This situation shows us that ABS has a positive effect on wound healing. In the comparison made between diabetes + control and diabetes + ABS groups, no significant difference was found, although a decrease in inflammation was detected. We think that the effect of ABS in diabetic wound healing should be investigated with further studies.

Many studies have reported that various cell sources contribute to the production of wound cytokines [28]. Previous studies examining the effect of IL-6 on the healing process have shown that IL-6 has a negative impact on wound healing by prolonging the colon wound healing process [29]. There are studies showing that IL-6 has a positive role in wound healing, suggesting that it speeds up the healing process in skin wound healing [30]. It has been suggested that TNF-α and IL-6 have negative effects on wound healing [31]. Studies have shown that adequate nitric oxide (NO) production is required for effective wound healing and topical application of NO accelerates the closure of excisional wounds in diabetic rats [32]. Also, impaired angiogenesis was established in mice with e-NOS deficiency [33]. In previous studies, it was reported that NO plays an important role in antimicrobial mechanisms by reducing bacterial loads in wound areas. It has also been suggested that when skin injury occurs, it forms a structure so that the injured tissue is not invaded by microbial pathogens [34].

In our study, when the IL-6 levels were evaluated, no significant difference was found between the groups on the 14th day. On the 21st day, a significant increase was observed in the IL-6 level in the ABS group compared to the control group. In the comparison between diabetes and diabetes + ABS, the first IL-6 levels were significantly lower in the ABS group. This shows that ABS has a positive effect on diabetic wound healing. On day 21, an increase in inflammation was observed in the CAPE group. On the 14th day of e-NOS, an increase was observed in both CAPE and ABS groups and it was found statistically significant. This shows us that both ABS and CAPE positively affect wound healing per day.

In conclusion, in this study, the effects of ABS and CAPE on the secondary wound healing in experimentally created excisional wounds on the gingival mucosa of diabetic and nondiabetic Wistar albino rats were evaluated by histological and PCR analysis methods. It is found that ABS and CAPE were successful as local agents in the nondiabetic group. The positive effects of ABS and CAPE in the early stage of wound healing were detected. ABS and CAPE can be useful in wound healing in periodontal and even oral surgery. It is necessary to shed light on the clinical effects on gingival wound healing by conducting studies on postoperative and long-term results following ABS and CAPE administration.
